# Smart Bioimpedance Device for the Assessment of Peripheral Muscles in Patients with COPD

**DOI:** 10.3390/s24144648

**Published:** 2024-07-17

**Authors:** David Naranjo-Hernández, Javier Reina-Tosina, Laura M. Roa, Gerardo Barbarov-Rostán, Francisco Ortega-Ruiz, Pilar Cejudo Ramos

**Affiliations:** 1 Biomedical Engineering Group, Department of Signal Theory and Communications, University of Seville, 41092 Seville, Spain; jreina@us.es (J.R.-T.); lroa@us.es (L.M.R.); gbarbarov@us.es (G.B.-R.); 2 Medical Surgical Unit of Respiratory Diseases, Virgen del Rocío University Hospital, 41013 Seville, Spain; francisco.ortega.sspa@juntadeandalucia.es (F.O.-R.); mariap.cejudo.sspa@juntadeandalucia.es (P.C.R.)

**Keywords:** bioimpedance, bioimpedance spectroscopy, biomedical device, COPD, muscle dysfunction, muscle atrophy, ultrasound image, isometric strength, isotonic strength

## Abstract

Muscle dysfunction and muscle atrophy are common complications resulting from Chronic Obstructive Pulmonary Disease (COPD). The evaluation of the peripheral muscles can be carried out through the assessment of their structural components from ultrasound images or their functional components through isometric and isotonic strength tests. This evaluation, performed mainly on the quadriceps muscle, is not only of great interest for diagnosis, prognosis and monitoring of COPD, but also for the evaluation of the benefits of therapeutic interventions. In this work, bioimpedance spectroscopy technology is proposed as a low-cost and easy-to-use alternative for the evaluation of peripheral muscles, becoming a feasible alternative to ultrasound images and strength tests for their application in routine clinical practice. For this purpose, a laboratory prototype of a bioimpedance device has been adapted to perform segmental measurements in the quadriceps region. The validation results obtained in a pseudo-randomized study in patients with COPD in a controlled clinical environment which involved 33 volunteers confirm the correlation and correspondence of the bioimpedance parameters with respect to the structural and functional parameters of the quadriceps muscle, making it possible to propose a set of prediction equations. The main contribution of this manuscript is the discovery of a linear relationship between quadriceps muscle properties and the bioimpedance Cole model parameters, reaching a correlation of 0.69 and an average error of less than 0.2 cm regarding the thickness of the quadriceps estimations from ultrasound images, and a correlation of 0.77 and an average error of 3.9 kg regarding the isometric strength of the quadriceps muscle.

## 1. Introduction

Muscle dysfunction is currently considered a systemic affectation that accompanies Chronic Obstructive Pulmonary Disease (COPD), whose importance lies in the impact it has on physical activity, effort capacity, quality of life and even survival of these patients [1,2,3,4,5,6,7,8]. Muscle weakness is normally accompanied by a loss of muscle mass compared to the healthy reference population, called muscle atrophy [5,9]. The prevalence rate of muscle atrophy in patients with COPD is in the range 4–35% [10,11], being more frequent in women and in more severe stages of the disease [10,11]. The muscle atrophy process in COPD mainly affects the muscles of the lower limbs [12,13,14]. The loss of muscle mass, evaluated by the fat-free mass index (FFMI), has also been observed in COPD patients with a normal body mass index (BMI), and up to a 26% decrease in muscle mass can be achieved [15].

These results highlight the need to investigate techniques and methods that allow the objective evaluation of muscle atrophy, which may be of interest not only in the diagnosis but also in the evaluation of the results obtained with the treatment or therapeutic interventions, such as muscle training applied in respiratory rehabilitation [14,16,17,18]. In the search for an objective marker of muscle deterioration, the cross-sectional area of the quadriceps ($A_{Q}$) [13,14,19,20] has been used. The decrease in this area, which can be evaluated by ultrasound or computed tomography, has been related to a worse prognosis of the disease [13,14,19,20].

**Figure 1:**
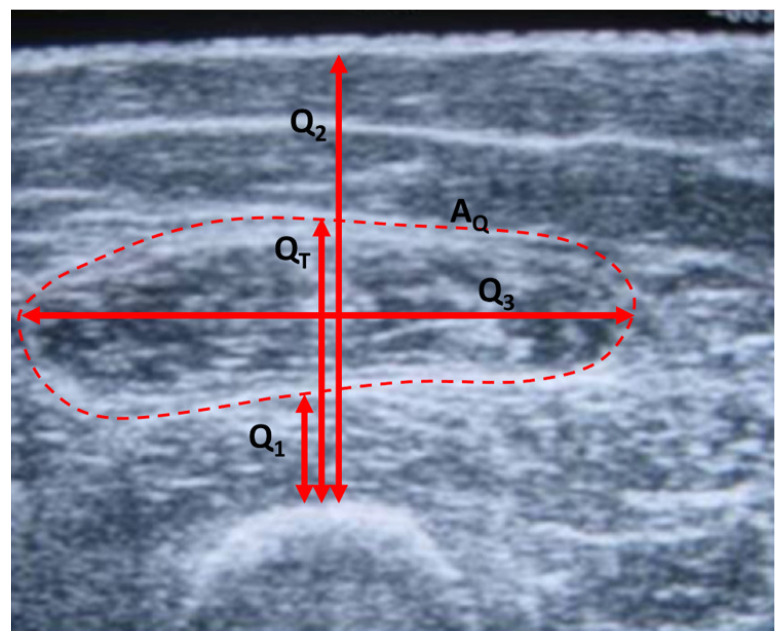
Thickness $Q_{T}$ and the area $A_{Q}$ on an ultrasound image of the quadriceps.

Another marker used in many studies to objectively evaluate muscle atrophy in the lower limbs is the thickness of the quadriceps ($Q_{T}$), calculated as the width of the muscle from the femur to the external fat surface, and measured at the midpoint of the line joining the knee to the greater trochant [21,22,23]. Figure 1 shows an example of $Q_{T}$ and $A_{Q}$ on an image of the quadriceps made by ultrasound. These parameters have been considered as indicators of the health status of patients with COPD [23,24] and predictors of complications and exacerbations [22,25].

The evaluation of muscle mass, in general, and the parameters $Q_{T}$ and $A_{Q}$, in particular, can be performed using medical imaging techniques such as computed tomography, magnetic resonance, dual-energy ray absorptiometry, or ultrasound, which allows for determining the thickness and cross-sectional area of muscles [21,22,23]. However, these techniques are expensive and must be performed and interpreted by specialized personnel, so they are not appropriate in routine clinical practice [14,21,26,27,28]. Bioimpedance measurements constitute a low-cost alternative to objectively evaluate muscle atrophy since they allow non-invasive measurement of the composition of the human body, discriminating between water, fat mass and muscle mass [14,26,27,28,29]. Its use in clinical practice is appropriate because the bioimpedance measurement equipment is small and portable, the measurements are easy to perform and interpret, without the need for specialized personnel, and they can be repeated as many times as desired [14,26,27,28,29]. Although the precision and clinical usefulness of bioimpedance when evaluating body composition has been proven in numerous studies [30,31], its application in peripheral muscle evaluation in COPD patients is still novel [14,26,28].

A recent work [32] has compared the quadriceps cross-sectional area in normal weight and obese older men with respect to segmental bioimpedance using characteristic frequency and phase angle parameters, but not with the typical bioimpedance parameters based on the Cole bioimpedance model (see Section 2). In [33,34] a correlation of muscle quality measured using ultrasound-derived echo intensity (EI) was found with respect to bioimpedance extracellular resistance in healthy young volunteers. However, EI does not provide estimations of dimensions or areas, but rather intensity levels obtained from gray-scale histogram analysis.

The objective evaluation of the muscle mass of the lower limbs must also be accompanied by a functional evaluation. A consensus established between the American Thoracic Society (ATS) and the European Respiratory Society (ERS) highlights the importance of functional muscular evaluation of the limbs in patients with COPD [35], and not only of its structural component. The different techniques for evaluating muscle strength (isokinetic, isometric, or isotonic) offer various evaluation perspectives, depending on the muscle group evaluated, the type of contraction, the speed of movement and the equipment used. In the aforementioned consensus, experts recommend the use of the isometric technique to measure muscle strength, since in this technique neither the angle of movement nor the length of the muscle changes [36]. However, isometric measurements of muscle strength performed with dynamometers must be supervised and interpreted by specialized health staff. Therefore, they are not commonly performed in routine clinical practice [36].

This work describes the adaptations made to the laboratory prototype of a bioimpedance spectroscopy measurement device [37,38] to be used in the muscle evaluation of patients with COPD. The main novelties of the work focus on the suitability of the device for measuring segmental bioimpedance in the quadriceps region, and the proposal of a set of equations based on the analysis of bioimpedance for the estimation of structural muscle parameters, such as $Q_{T}$ and $A_{Q}$, but also functional ones, such as isometric and isotonic force. These are differential and novel aspects of the work, since to the authors’ knowledge, bioimpedance is normally applied to the evaluation of body composition (water, fat mass and fat-free mass) with full body measurements [14,26,28] or segmental measurements of the entire leg [27], but never in the estimation of structural or functional parameters of localized muscles. The bioimpedance device and the proposed equations for estimating muscle parameters have been validated through a pseudo-randomized study in patients with COPD in a controlled clinical environment, contrasting the estimations with measurements from ultrasound images for structural parameters, and evaluations of isometric and isotonic strength performed by dynamometers for functional parameters.

## 2. Materials and Methods

The custom-made laboratory prototype of the bioimpedance spectroscopy device has been adapted through a user-centered design methodology, according to which the opinions, requirements and improvements suggested by users, both clinicians and patients, were collected through a series of questionnaires and semi-structured interviews, and were finally incorporated into the design process. For the research and development of the device, an iterative development cycle carried out in parallel to the validation has been used. One of the bases of this methodology is the application of the concept of functional and structural modularity, according to which the device is made up of a series of modules with differentiated functions and interconnected with each other with well-defined interfaces, which favors re-design and updating components in the iterative development cycle. Regarding the starting prototype [37], the adaptation of the bioimpedance device has involved the implementation of a new sensorization stage (cables, electrodes, current injection and measurement, described in more detail in Section 3.1), the adaptation of the firmware for data processing and estimation of bioimpedance parameters in quadriceps measurements, as well as a modification of wireless communications for data transmission.

After the bioimpedance device was technically validated, a pseudo-randomized study was carried out in patients with COPD whose objective was the comparative analysis of bioimpedance parameters in the quadriceps region, with respect to morphological evaluations using ultrasound, $Q_{T}$ and $A_{Q}$, and assessments of the isometric and isotonic strength of such muscles. The characteristics of the study are summarized next:

- **Study population**: Patients diagnosed with COPD following the criteria established by the American Thoracic Society (ATS) and the Spanish Society of Pulmonology and Thoracic Surgery (SEPAR) who presented moderate-severe airflow obstruction (forced expiratory volume in the first second ($FEV_{1}$ ) < 80%) and with a clinical impact of their disease (criteria for inclusion in the respiratory rehabilitation program of the Virgen del Rocío University Hospital in Seville, Spain). All subjects gave their informed consent for inclusion before they participated in the study. The study was conducted in accordance with the Declaration of Helsinki, and the protocol was approved by the Research Ethics Committee of the Virgen Macarena and Virgen del Rocío University Hospitals (Project identification code: PI-0041-2014). The patients were stable and with appropriate therapy, and had not suffered exacerbations of the disease in a period of three months prior to the study, and without treatment with oral corticosteroids for at least the same period.
- **Measurements**: The reference measurements of muscle dimensions were carried out by ultrasound using an Aloka SSD-900 Ultrasound Machine (ALOKA Co. Ltd., Tokyo, Japan). Procedure: The patient was placed in a supine position, with a rolled towel under the popliteal fossa to relax the thigh. The transducer was placed with the long axis perpendicular to the longitudinal axis of the rectus femoris muscle, at the midpoint between the anterior superior iliac spine and the superior edge of the patella. Using the device’s software, the area and dimensions of different muscle sections were estimated, recording the data for subsequent analysis. The following measurements were obtained, referring to the image in Figure 1: thickness of the vastus intermedius ($Q_{1}$), length from the femur to the upper limit of the rectus femoris ($Q_{T}$), length from the femur to the skin ($Q_{2}$), transverse thickness of the rectus femoris ($Q_{3}$) and area of the rectus femoris ($A_{Q}$). Isometric strength is defined as a static contraction without a change in muscle length. To measure this strength, the subject was seated and with a knee flexion of 90°. The knee was extended against static resistance and the isometric quadriceps strength $F_{isoM}$ was established using an isometric dynamometer. The isotonic force $F_{isoT}$ was evaluated through the 1RM test, defined in this case as the maximum weight that an individual sitting and with knee flexion of 90º can lift in a single repetition. For bioimpedance measurements, the adapted device described in the present work was used following the measurement protocol described in Section 2.1.
- **Data analysis**: For the comparative analysis of the groups, the mean value and standard deviation (SD) were used. To compare the means between groups, the one-factor analysis of variance (ANOVA) method was used. Normality was established by the D’Agostino and Pearson test. The reference values used in the correlation analysis were estimations of quadriceps dimensions performed by ultrasound and measurements of muscle strength (isometric and isotonic). The reference values were compared with the parameters of the Cole bioimpedance model using the Pearson coefficient (or the Spearman coefficient in case the data did not have a normal distribution), considering results statistically significant when p<0.05. The regression line that minimized the mean square error was estimated, performing a Bland–Altman agreement analysis, calculating the average difference between the measurements and the predictions of the regression line, with its corresponding SD. According to the Bland–Altman method, the 95% confidence intervals of the differences were calculated from the upper and lower agreement limits, (±1.96 times the SD). The Matlab desktop environment (version R2022a) for Windows was used to analyze the data.

### 2.1. Experimental Setup for Bioimpedance Measurements

**Figure 2:**
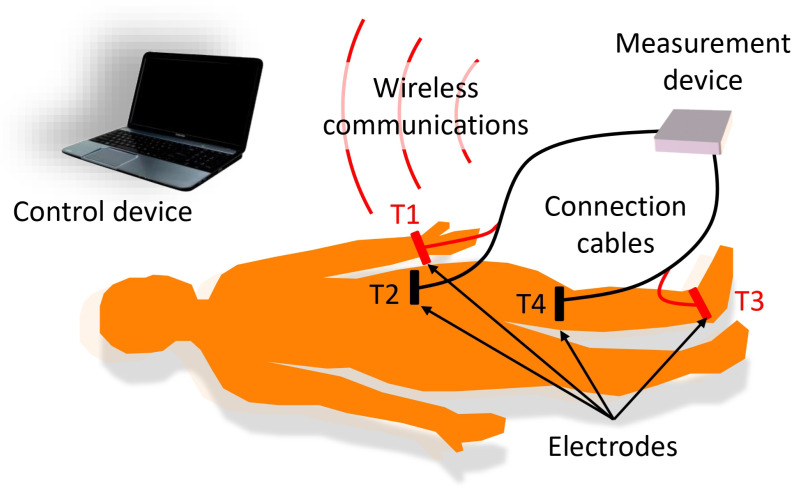
Setup scheme for bioimpedance measurements in the quadriceps region.

**Figure 3:**
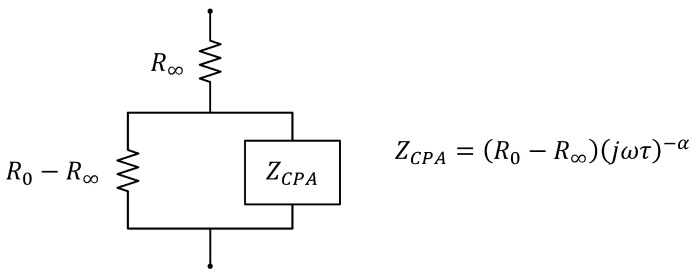
Equivalent circuit of the Cole model of bioimpedance [39].

To carry out the bioimpedance measurements, the following procedure was performed:

1. Before carrying out the measurements, the patient remained on a stretcher in a supine position for 5 min to promote homogeneous distribution of water throughout the body.
2. For the bioimpedance measurements, four electrodes were used, following the measurement scheme described in [37], with two electrodes for current injection (T1 and T3 in Figure 2) and two electrodes for voltage measurement (T2 and T4 in Figure 2). The current injection electrodes were placed on the foot (dorsal surface at the level of the metatarsals) and the hand (dorsal surface above the knuckles) on the side of the body corresponding to the leg on which the measurements were made. The voltage measurement electrodes were located at the ends of the quadriceps region, one above the kneecap and the other at the level of the anterior superior iliac spine. Figure 2 shows a scheme of the measurement procedure and the location of the electrodes.
3. In each measurement, the device estimated the real and imaginary part of the bioimpedance in 22 frequencies distributed logarithmically between 5 kHz and 1 MHz.
4. The complex bioimpedance values were sent wirelessly to a laptop computer (control device, in the modular scheme described in Section 3.1), which estimated the parameters of the extended-Cole bioimpedance model that best fit with the bioimpedance values (see Table 1):
  The equations that define the extended-Cole model are the following, with ω being the angular frequency that corresponds to 2πf and *f* the frequency in Hz:
  (1) $$Z=\left(R_{\infty }+\frac{R_{0}-R_{\infty }}{1+{\left(j\omega \tau \right)}^{\alpha }}\right)e^{j\omega T_{D}}$$
  (2) $$R_{ECW}=R_{0}$$
  (3) $$R_{ICW}=\frac{R_{0}\cdot R_{\infty }}{R_{0}-R_{\infty }}$$
  (4) $$C_{M}=\frac{\tau }{\left(R_{ICW}+R_{ECW}\right)}$$
  According to [39], the equivalent circuit of Equation (1) without taking into account $T_{D}$ corresponds to the diagram shown in Figure 3, which includes a constant-phase-angle impedance $Z_{CPA}$.
5. The measurement process was repeated 3 times sequentially to allow the analysis of the repeatability of the measurements. Finally, the average value of the bioimpedance parameters of the three measurements was selected for the correlation and agreement analysis.

## 3. Results

### 3.1. Prototype Design and Development

**Figure 4:**
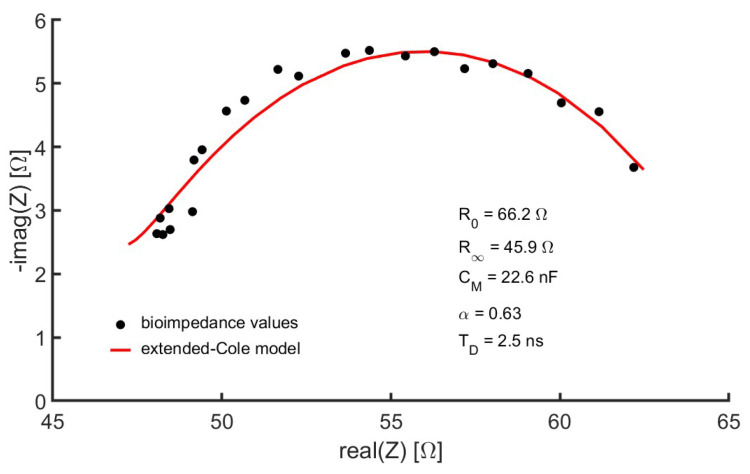
Example of bioimpedance values in a quadriceps measurement.

The device presented in [37] was modified to allow bioimpedance measurements to be performed in the quadriceps region. In the context of this work, the device is considered a smart sensor since not only has sensorization capabilities for measuring certain variables but is also equipped with a communications unit and the capacity to carry out pre-processing of the acquired sensing information [40]. The modular architecture of the device facilitated the adaptation process. Figure 2 also highlights the elements of this modular architecture. Following the proposed modular scheme, the main adaptations were the following:

- **Electrodes**: The electrodes validated in [37] were also used for measurements in the quadriceps region for the following reasons: being adhesive, movement artifacts are avoided; the conductive gel in contact with the skin and the Ag/AgCl chemical electrode reduces the impedance of the electrode-skin contact; the rectangular surface of 6 cm × 1.5 cm, with the shorter length in the current circulation line, reduces the impedance of the electrodes compared to standard solutions based on circular electrodes with a smaller contact surface.
- **Measurement device**: The device was battery-powered and bioimpedance measurements were based on the spectroscopy technique to allow differentiation between the extracellular and intracellular compartments. In each bioimpedance measurement, the module and phase of the bioimpedance were measured sequentially at the frequencies mentioned in Section 2. The measurement process lasted 25 s following the functional scheme described in [37]. The injected current was set at 0.4 mA to avoid any type of damage to the human body. The instrumentation amplifier was modified to obtain a gain of 26 dB in the measured voltage, a value chosen to provide the best signal-to-noise ratio considering that the impedance of the quadriceps is much lower than that pre-established for the whole body, and with sufficient robustness so that the signals do not saturate in any volunteer. To estimate the module and phase at the different frequencies, the quadrature signal demodulation method was used following the procedure described in [37].
- **Connection cables**: The electrodes were attached to the measurement device by two 140 cm-length cables, which provided sufficient ergonomics and flexibility to loosely arrange the measurement device in an area close to the measurement subject. Two isolated active lines were inside each cable, one for connection to a current injection electrode and another for connection to a voltage measurement electrode. The lines were twisted to minimize mutual interference and shielded on the outside to remove external interference. In the last 30 cm, the lines were separated to facilitate the connection to the electrodes, which in turn, was carried out by using self-adjusting crocodile-type clips.
- **Control device**: A laptop computer served as the user interface for controlling the measuring device. This interface was programmed in the MATLAB environment (version R2022a) from GUI applications. Healthcare professionals helped establish GUI specifications based on requirements specified from interviews and questionnaires. The interface allowed the recording of the anthropometric parameters of the subject under study, such as weight, age, sex, height and external dimensions of the measurement area, taken with a flexible measuring tape: distance between the voltage measurement electrodes, perimeter of the leg at the point where the reference ultrasound-based measurements were performed (see Section 2). A button on the interface started the measurement process. The values of the module and phase of the bioimpedance at the different frequencies were converted into a representation in the complex plane. Using genetic algorithms, the parameters of the extended-Cole model that minimized the error with respect to the bioimpedance values were estimated and also displayed in the GUI. In this work, an implementation of the classical genetic algorithms [41,42] with a population of 10,000 and 12 generations has been used. The bioimpedance values and the processing results were stored in files with the date and time of measurement. Figure 4 shows an example of bioimpedance values in one of the measurements made on the quadriceps to one of the volunteers, along with the extended-Cole model that best fits the bioimpedance values.
- **Wireless communications**: A bidirectional wireless communications link was established between the measuring device and the control device. A protocol was developed for sending commands from the control device, including the measurement start command, and sending data from the measurement device in a sequence of information triplets (module, phase and frequency). To implement communications, the Microchip RN42 transceiver (Microchip Technology Inc., Chandler, Arizona, USA) was used, developing an SPP serial interface over the Bluetooth v2.1 standard.

### 3.2. Technical Evaluation

As the length of the connecting cables introduces a delay in the signal and the gain of the amplifiers is altered at high frequencies due to the available bandwidth, but also at low frequencies (the module and phase estimation method using the demodulation of quadrature signals uses a low-pass filter that alters the results at low frequency), it was necessary to perform a calibration that allowed correcting the deviations in both module and phase as a function of frequency.

**Figure 5:**
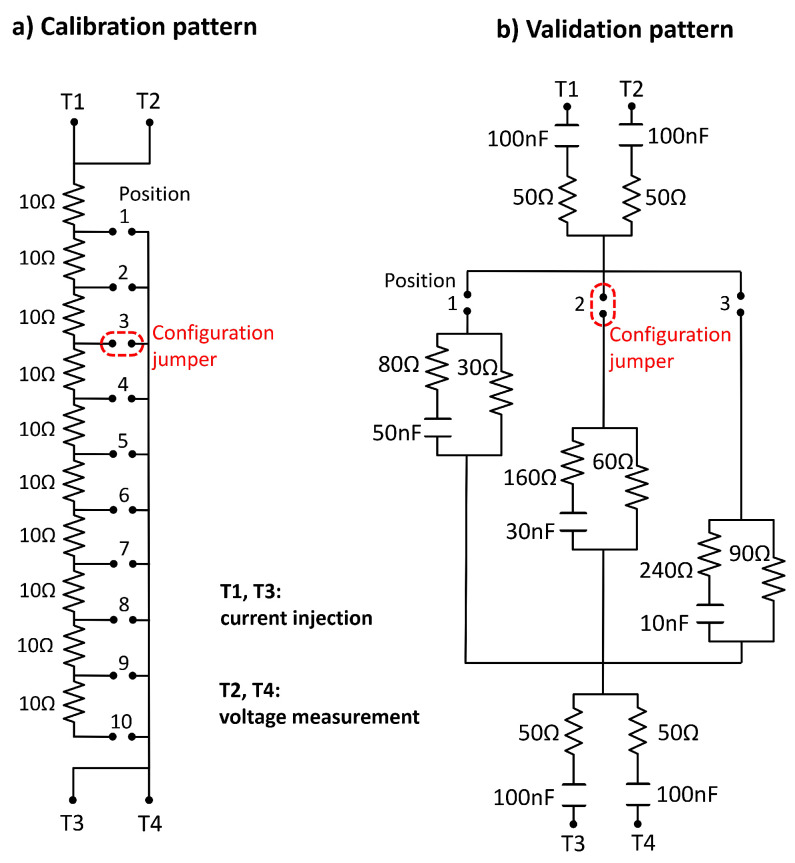
Calibration pattern (**a**) and validation pattern (**b**) used in the technical validation of the device.

For this new calibration, the calibration pattern shown in Figure 5a was used, performing a frequency sweep in 10 different positions of the pattern, from 10Ω to 100Ω, in increments of 10Ω. The differences obtained in the module and phase with respect to the expected values in each of the positions of the calibration standard allow the establishment of the necessary corrections that must be made depending on the frequency.

Once the device was calibrated, it was technically validated by using a circuit pattern adjustable in three positions (see Figure 5b). In each of the positions of the validation pattern, a circuit is established consisting of a branch that models the extracellular path of current circulation through a resistance, and another branch in parallel that models the intracellular path through a resistance in series with a capacitance. This circuit has a frequency behavior similar to that found in bioimpedance measurements in the quadriceps region. The values of the resistances and the capacitance in the three positions were set to represent the typical bioimpedance values in the quadriceps region in three cases: minimum, average and maximum bioimpedance. These bioimpedances were initially evaluated in a preliminary study in different volunteers and subsequently corroborated in the clinical validation study of the device.

Both the calibration and validation patterns have four terminals for connection to the device thanks to the clamps at the ends of the connection cables. Two terminals on the complementary sides are used for current injection, while the other two adjacent terminals are used for voltage measurement. The validation pattern also includes a simple electrode circuit model on each of the terminals to provide a better approximation of the conditions of use of the device. To model the behavior of the electrodes, a series circuit was used formed by a capacitor of 100 nF, which reproduces the double layer effects of the electrode-skin contact, and a 50-Ω resistor, which represents the resistance of the electrolyte gel. These values were functionally approximated by taking into account the surface of the electrodes based on the experimental results presented in [43].

Multifrequency bioimpedance measurements were carried out on the validation pattern in its three positions, and the error committed in all cases was always lower than 1% with respect to the expected value according to the circuit analysis, which allowed the bioimpedance device to be technically validated.

### 3.3. Clinical Evaluation

Once the device was technically validated, a pseudo-random study of correlation and agreement of bioimpedance measurements with ultrasound parameters of the quadriceps muscle region, and evaluations of the isometric and isotonic strength of these muscles, was carried out. In this preliminary evaluation study, 33 patients with COPD participated, 7 women (group I) and 26 men (group II), from whom measurements of quadriceps dimensions were taken according to the procedure described in Section 2. A subgroup of volunteers (group III), consisting only of men, also underwent evaluations of isometric strength and isotonic strength. Table 2 summarizes the characteristics of the volunteers participating in the study.

For each of the measurements made on the volunteers, the parameters of the Cole bioimpedance model that best approximated the experimental measurements were calculated according to the mean square error. $T_{D}$ parameter is not included due to its main dependence on hardware and cable length. Table 2 shows the mean value and SD of the bioimpedance parameters in each of the groups, and globally. On the other hand, Table 3 shows the results of the repeatability analysis of the bioimpedance measurements, for which the mean and SD of the mean absolute error, and the mean and SD of the mean absolute relative error, both calculated with the bioimpedance parameters obtained in successive measurements, are used.

A correlation study of these parameters was carried out with respect to ultrasound estimates of quadriceps muscle dimensions and isometric and isotonic strength evaluations with dynamometers.

Table 4 shows the results of the correlation analysis with respect to parameter $Q_{T}$ for each of the groups (women, men and the strength study subgroup), and for the global set of users. The use of the inverse of the resistive parameters of the Cole model must be taken into account, according to the dependence of the impedance values on the dimensions of the transverse surface, thus obtaining a positive correlation.

All bioimpedance parameters except α show some degree of correlation with $Q_{T}$. The bioimpedance parameter that shows the greatest correlation with the parameter $Q_{T}$ estimated by ultrasound is $C_{M}$, associated with effective membrane capacitance. The correlation found with $C_{M}$ is above 0.68 in all the groups studied. The results are corroborated by the value of statistical significance, which is often much lower than 0.05, except in group I (women). The worsening of statistical significance in this group may be related to the low number of available measurements.

**Figure 6:**
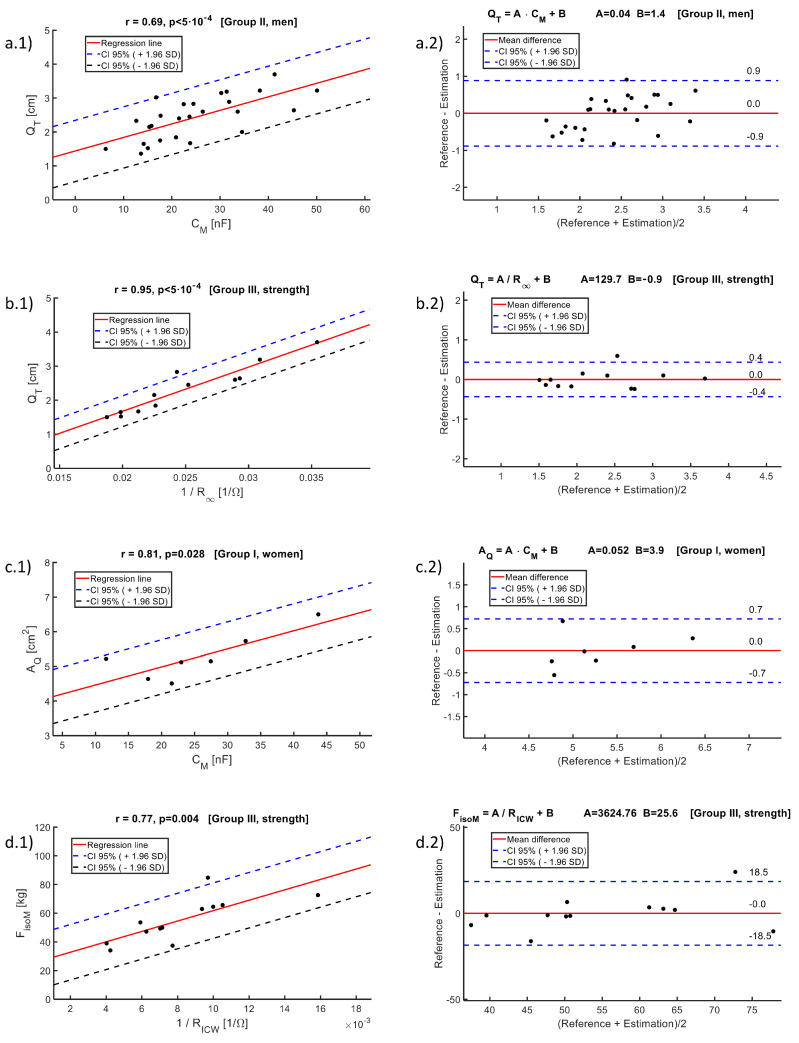
(**a**) Correlation of $Q_{T}$ with respect to $C_{M}$ in Group II (men). (**b**) Correlation of $Q_{T}$ with respect to $1/R_{\infty }$ in Group III (strength subgroup). (**c**) Correlation of $A_{Q}$ with respect to $C_{M}$ in Group I (women). (**d**) Correlation of $F_{isoM}$ with respect to $1/R_{ICW}$ in Group III (strength subgroup). (**x.1**) Correlation graph. (**x.2**) Bland–Altman’s analysis based on the regression estimations.

Figure 6 summarizes some of the best correlations found between bioimpedance parameters and structural and functional estimations of the quadriceps muscle. Figure 6 shows an example of correlation graphs between $Q_{T}$ and $C_{M}$, since this is the parameter that has shown the greatest correlation with the quadriceps structural parameters, as well as the agreement results according to the Bland–Altman analysis. The estimations used for such analysis correspond to the prediction equations of $Q_{T}$ from $C_{M}$ described in Table 5, which were obtained through a linear regression model. Table 5 also shows some statistics of the error associated with the estimations, such as the mean error (ME) or the mean square error (MSE), which does not exceed 0.2 cm. To allow a comparative framework between different Bland–Altman diagrams, the ordinate axis of the graphs has been sized to allow visualization of a maximum error corresponding to the difference between the maximum and the minimum in the reference values.

Another of the markers used as indicators of the health status of patients with COPD is $A_{Q}$. Table 4 also summarizes the results of the correlation analysis of this parameter with respect to the different bioimpedance parameters obtained after the identification of the extended-Cole model. In this case, the correlation has decreased when compared to the results obtained with $Q_{T}$. As also shown in Table 4, the parameter for which a greater correlation and a better statistical significance is obtained is $C_{M}$. Similarly, Figure 6 shows an example of a correlation graph between $A_{Q}$ and $C_{M}$, as well as the agreement results according to the Bland–Altman analysis based on the estimations made with the prediction equations of $A_{Q}$ with respect to $C_{M}$ that are described in Table 5. In this case, the mean square error is around $1\;{\mathrm{cm}}^{2}$, in estimations of $A_{Q}$ that have a mean value of $5.3\;{\mathrm{cm}}^{2}$ (see Table 2).

Although the parameters $Q_{1}$, $Q_{2}$ and $Q_{3}$ are not usually used in the literature as indicators of the health status of patients with COPD, these parameters are also included in the present work to provide additional information on the structural components of the quadriceps muscle. In this way, Table 4 shows the results of the correlation analysis of the parameters $Q_{1}$, $Q_{2}$ and $Q_{3}$, respectively, with respect to the bioimpedance parameters, and for each of the study groups. Except in Group I, the bioimpedance parameters related to resistive values and membrane capacity also show good correlations with $Q_{1}$ and $Q_{2}$. This result was expected since these parameters correspond to other structural parameters of the quadriceps muscle similar to the dimensions described by $Q_{T}$ and $A_{Q}$. In Group I, $C_{M}$ is the parameter that shows the highest correlation, but it does not have sufficient statistical significance. As previously mentioned, this fact may be due to the small number of measurements in this group. It is also worth noticing the high correlation found in the parameter $Q_{2}$ with respect to τ in Group I, 0.84, with statistical significance. On the other hand, there is a remarkable lack of correlation of the bioimpedance parameters with the parameter $Q_{3}$, which has been obtained in the direction transverse to $Q_{1}$ and $Q_{2}$ as can be seen in Figure 1.

The functional components of the quadriceps muscle were established by the isometric strength $F_{isoM}$ and the isotonic force $F_{isoT}$. Table 4 summarizes the results of the correlation analysis of both strengths with respect to the bioimpedance parameters for the measurements of Group III. The results obtained indicate a higher correlation of the bioimpedance parameters with the functional components than with the structural components. In this case, the parameter that showed the highest correlation, and the highest statistical significance, was $1/R_{ICW}$, with a correlation of 0.70 for $F_{isoT}$, and 0.77 for $F_{isoM}$.

Figure 6 shows the correlation graph of the parameters corresponding to $F_{isoM}$ with respect to $1/R_{ICW}$ for Group III, as well as the agreement results according to the Bland–Altman analysis based on the estimations made with the prediction equations described in Table 5. In this case, the mean error is around 3.9kg in estimations of $F_{isoM}$ with an average value of 55.1kg (see Table 2).

Figure 6 also shows the best correlation obtained for the parameter $Q_{T}$ in Group III, in this case with respect to $1/R_{\infty }$, exhibiting a correlation of 0.95 and an error average of 0.1cm.

## 4. Discussion

Numerous studies have shown the clinical usefulness of evaluating the peripheral muscles using ultrasound for the assessment of muscle dysfunction and muscle atrophy in patients with COPD [14,16,17,18]. Among the most prominent markers are the thickness of the quadriceps $Q_{T}$, mainly, and the cross-sectional area of the quadriceps $A_{Q}$ [21,22,23]. This evaluation is not only of interest in the diagnosis of muscle dysfunction but also in the monitoring of therapeutic interventions such as muscle training applied in respiratory rehabilitation [22,23,24,25].

However, ultrasound imaging technique has certain limitations for its use in routine clinical practice, since it must be performed and interpreted by specialized healthcare personnel [21]. Furthermore, the magnitudes and dimensions obtained by ultrasound have a certain degree of subjectivity, since they are dependent on the operator and the measurement context to the extent that the pressure exerted on the probe or the positioning and inclination of the probe on the skin can significantly affect the evaluations. Additionally, the evaluation of intermuscular septae may be difficult to perform in elderly subjects or in those with tissue depletion [21].

In this work and in the search for an alternative to the objective evaluation of the peripheral muscles, the bioimpedance technique has been proposed due to its numerous practical advantages, especially its ease of use, low-cost, high inter- and intra-observer reproducibility, and its safety that allows the measurements can be repeated as many times as desired. To allow segmental measurement of bioimpedance in the quadriceps region, the device proposed in [37] was adapted following a user-centered design methodology, focused mainly on the implementation of a new sensorization stage that allows localized measurement in the quadriceps region and a hardware calibration that minimizes errors in the estimation of the parameters of the Cole model that fit the bioimpedance values.

Although this is a preliminary study, an estimation of the bioimpedance device performance can be performed by comparing the mean absolute error of the bioimpedance parameters in successive measurements given in Table 3 with respect to the SD of those same parameters in the global group of volunteers in Table 2. For such estimation, the mean absolute error in successive measurements can be considered as device precision and the dynamic range of bioimpedance parameters in the quadriceps region can be considered as approximately four times the SD (confidence interval). In that case, $R_{0}$ is the most accurate parameter, since there would be 208 possible values in its dynamic range. $R_{\infty }$ would be the second most precise parameter, with 158 possible values in its dynamic range. $C_{M}$ would allow 42 possible values, and the least precise would be α, with 32 possible values. On the other hand, it should be noted that the bioimpedance parameters of the quadriceps muscle were very similar in all groups, with some differences, although not significant between men and women at $R_{0}$ (p=0.059 according to ANOVA analysis).

According to the results shown in the present work, a close correlation and correspondence have been found between the bioimpedance parameters obtained in segmental measurements in the quadriceps region with respect to structural parameters related to the thickness of the quadriceps muscle, from the bone to the skin in its different structures ($Q_{1}$, $Q_{2}$, $Q_{T}$, and even $A_{T}$), but not in other structural components of a transversal nature, such as the parameter $Q_{3}$. Perhaps this is the reason why a greater correlation of the bioimpedance parameters is obtained with respect to the parameter $Q_{T}$, reaching the value of 0.69 in the global case, than with respect to the parameter $A_{T}$, for which a correlation of 0.45 is obtained in the global case.

The bioimpedance parameter that has shown the best correspondence with the structural parameters obtained by ultrasound has been $C_{M}$. Although the physical meaning and origin of $C_{M}$ is still unclear [31,44], since there are many effects that can influence (passive cell membrane capacitance, Maxwell–Wagner effects, intracellular organelle membranes, protein molecule response) [45], some authors relate it to the cell membrane capacitance [46,47,48], sometimes incorporating the effect of other phenomena, such as the capacitance of intercellular connection regions [44].

Good correlations are also obtained with the inverse of $R_{ICW}$, a parameter related to intracellular resistance. This relationship is also expected since $R_{ICW}$ will decrease as muscle cell mass increases; therefore, its inverse will increase. This effect is also observed in the inverse of $1/R_{\infty }$, but to a lesser extent in the inverse of $1/R_{ECW}$, since $R_{\infty }$ would be the equivalent resistance of the parallel of $R_{ECW}$, which models the extracellular path of the electric current, and $R_{ICW}$, which models the intracellular path.

On the other hand, clinical guidelines highlight the importance of functional evaluation of muscle mass in the lower limbs, not only the structural components, measured through isometric or isotonic strengths [35,36]. Both assessments, structural and functional, are related and numerous studies have shown a causal relationship between the parameters $Q_{T}$ and $A_{Q}$ estimated by ultrasonography with respect to the strength capacity of the quadriceps muscle in patients with COPD [14,19,49], seniors [24,50], but also in young healthy people [51]. In this work, the relationship of the bioimpedance parameters obtained from the quadriceps region with respect to the expression of both strengths, isotonic and isometric, has also been analyzed, finding an even greater correlation, with a value of 0.70 for the isotonic strength, but reaching a value of 0.77 for isometric strength. These results may be related to the problems of subjectivity and inter- and intra-observer dependence of muscle estimations performed by ultrasound. As bioimpedance parameters provide greater correspondence with respect to the functional parameters of the muscle, perhaps bioimpedance, which eliminates subjective inter- and intra-observer effects, can provide more objective measurements of muscle than ultrasound itself.

The linear relationship of the muscle parameters (both structural and functional) observed with respect to the resistive parameters of the Cole bioimpedance model is consistent with what was expected since it is foreseeable that a larger muscle size will increase electrical muscle resistance in the same way, both extracellular and intracellular, but also at zero frequency or an infinite frequency. On the other hand, as $C_{M}$ can be related to the effective capacitance of muscle, a linear relationship with the functional and structural parameters of the quadriceps muscle is also expected.

In [52] it was established that ultrasound is superior to bioimpedance spectroscopy in muscle mass measurements according to the results of [53]. However, this study was conducted in critically ill patients using muscle mass estimations provided by two commercial devices: Bioscan 920-2 (Malton International, Malton, UK), which performs multi-frequency analysis at 5, 50, 100, and 200 kHz, and Physion MD (Nippon Shooter Ltd., Tokyo, Japan), based on single-frequency analysis at 50 kHz. The results of the present study have been obtained using the bioimpedance spectroscopy technique, which provides higher precision than single-frequency and multi-frequency techniques [30], and estimations of bioimpedance parameters directly related to the bioelectric behavior of the muscle region, not muscle mass estimations that derive from particular models internally calculated in commercial devices.

The main limitation of the study is the low number of measurements, being very small in the study group of women. However, the results of correlation and agreement between the bioimpedance parameters and the structural and functional evaluations of the quadriceps muscle provide important evidence on the usefulness and encourage continuing with the research study and development of a bioimpedance device for the evaluation of peripheral muscles in patients with COPD.

As an added value of the study, a series of prediction equations for the structural and functional parameters based on the bioimpedance parameters are provided. Furthermore, it should be noted that in this work the gender perspective has been taken into account, by carrying out differential studies for men and women. This differentiation must be taken into account as a consequence of gender-related differences in the clinical characteristics, treatment, quality of life and costs of COPD [54,55,56].

## 5. Conclusions

A multifrequency bioimpedance device has been adapted for use in the evaluation of the peripheral muscles in patients with COPD, specifically the quadriceps muscle, due to its importance in the diagnosis, prognosis and monitoring of the disease, as well as therapeutic interventions such as muscle training applied to respiratory rehabilitation. The validation results confirm the correspondence and agreement of the bioimpedance parameters with the structural evaluations of the quadriceps muscle using ultrasound, and functional evaluations using isometric and isotonic strength tests.

The best correspondence results obtained for the functional parameters suggest that bioimpedance can solve the subjectivity problems presented by ultrasound evaluations due to intra- and inter-observer variability.

Although correspondence has been found with the resistive parameters of bioimpedance, the greatest correlation and correspondence with the functional and structural parameters of the quadriceps muscle has been found with respect to the capacitance of the membrane.

It has been possible to establish a series of prediction equations for the functional and structural parameters of the quadriceps muscle, for a global case, but also personalized according to gender.

The results obtained encourage to continue the research and proposal of bioimpedance as an objective measurement of peripheral muscles in patients with COPD for better diagnosis and monitoring and its routine clinical application.

## Figures and Tables

**Table 1:** Parameters of the extended-Cole model obtained from the complex bioimpedance values.

Parameter	Interpretation	Units
$R_{0}$	Resistance at zero frequency	Ω
$R_{\infty }$	Resistance at infinite frequency	Ω
$R_{ECW}$	Extracellular resistance	Ω
$R_{ICW}$	Intracellular resistance	Ω
τ	Time constant that characterizes the frequency relaxation distribution	seconds (s)
$C_{M}$	Effective membrane capacitance	Farads (F)
	(the physical meaning of this parameter is discussed in Section 4)	
α	Characteristic order of frequency relaxation distribution	dimensionless
$T_{D}$	Phase delay due to hardware and cable length	seconds (s)

**Table 2:** Characteristics of the volunteers participating in the study. Mean value ± SD.

	Group I, Women	Group II, Men	Group III, Strength	Global
Number of volunteers	7	26	12	33
Weight (kg)	76.3 ± 20.5	84.7 ± 17.2	79.5 ± 14.5	82.9 ± 18.3
Age (years)	56.4 ± 8.5	64.6 ± 7.2	66.3 ± 5.7	62.8 ± 8.2
Height (cm)	157.1 ± 6.9	167.8 ± 7.1	166.8 ± 5.8	165.5 ± 8.3
Body mass index ($\mathrm{kg}/m^{2}$)	30.8 ± 7.1	30.0 ± 5.6	28.4 ± 4.5	30.2 ± 6
$Q_{T}$ (cm)	2.5 ± 0.5	2.4 ± 0.6	2.3 ± 0.7	2.5 ± 0.6
$Q_{1}$ (cm)	1.2 ± 0.3	1.2 ± 0.4	1.1 ± 0.3	1.2 ± 0.4
$Q_{2}$ (cm)	3.8 ± 0.5	3.2 ± 0.8	2.9 ± 0.7	3.3 ± 0.8
$Q_{3}$ (cm)	4.7 ± 0.5	4.8 ± 0.8	4.5 ± 0.6	4.8 ± 0.8
$A_{Q}$ (${\mathrm{cm}}^{2}$)	5.3 ± 0.6	5.3 ± 1.7	5.0 ± 2.0	5.3 ± 1.5
$F_{isoT}$ (kg)	-	-	27.8 ± 6.3	-
$F_{isoM}$ (kg)	-	-	55.1 ± 14.7	-
$R_{0}$ (Ω)	72.6 ± 17.4	60.9 ± 13.1	61.3 ± 11.6	63.3 ± 14.6
$R_{\infty }$ (Ω)	47.4 ± 12.1	41.6 ± 9.1	41.6 ± 8.0	42.9 ± 9.9
$C_{M}$ (nF)	25.4 ± 10.4	24.7 ± 10.9	23.9 ± 11.2	24.9 ± 10.7
α (dimensionless)	0.62 ± 0.07	0.61 ± 0.09	0.59 ± 0.11	0.62 ± 0.08

**Table 3:** Errors obtained in the repeatability study in successive bioimpedance measurements: Mean value ± SD.

	Mean Absolute Error	Mean Absolute Relative Error
$R_{0}$ (Ω)	0.3 ± 0.2	0.4% ± 0.3%
$R_{\infty }$ (Ω)	0.3 ± 0.2	0.6% ± 0.5%
$C_{M}$ (nF)	1.0 ± 0.8	3.9% ± 2.7%
α (dimensionless)	0.01 ± 0.01	2.1% ± 1.8%

**Table 4:** Results of the correlation analysis of the bioimpedance parameters with respect to $Q_{T}$, $Q_{1}$, $Q_{2}$, $Q_{3}$ and $A_{Q}$: *r* (*p*), with *r* being the correlation coefficient and *p* being the statistical significance. Results with a correlation greater than 0.5 or a statistical significance less than 0.05 have been bolded.

Reference	Parameter	Units	Group I, Women	Group II, Men	Group III, Strength	Global
			r (p)	r (p)	r (p)	r (p)
	$1/R_{0}$	1/Ω	0.11 (0.813)	**0.66** (<$5\cdot 10^{-4}$)	**0.74** (**0.006**)	0.47 (**0.006**)
	$1/R_{\infty }$	1/Ω	0.11 (0.814)	**0.78** (<$5\cdot 10^{-4}$)	**0.95** (<$5\cdot 10^{-4}$)	**0.63** (<$5\cdot 10^{-4}$)
$Q_{T}$ (cm)	$1/R_{ICW}$	1/Ω	**0.54** (0.214)	**0.68** (<$5\cdot 10^{-4}$)	**0.88** (<$5\cdot 10^{-4}$)	**0.65** (<$5\cdot 10^{-4}$)
	$C_{M}$	*F*	**0.68** (0.090)	**0.69** (<$5\cdot 10^{-4}$)	**0.79** (**0.002**)	**0.69** (<$5\cdot 10^{-4}$)
	τ	*s*	**0.51** (0.241)	0.41 (**0.039**)	0.34 (0.280)	0.42 (**0.015**)
	α	-	0.36 (0.429)	0.09 (0.651)	0.09 (0.781)	0.03 (0.867)
	$1/R_{0}$	1/Ω	0.29 (0.533)	**0.64** (<$5\cdot 10^{-4}$)	**0.64** (**0.024**)	0.42 (**0.014**)
	$1/R_{\infty }$	1/Ω	0.10 (0.817)	**0.67** (<$5\cdot 10^{-4}$)	**0.79** (**0.002**)	**0.51** (**0.002**)
$Q_{1}$ (cm)	$1/R_{ICW}$	1/Ω	0.31 (0.493)	0.49 (**0.011**)	**0.71** (**0.009**)	0.46 (**0.007**)
	$C_{M}$	*F*	0.47 (0.284)	**0.55** (**0.004**)	**0.67** (**0.018**)	**0.53** (**0.001**)
	τ	*s*	0.42 (0.346)	0.33 (**0.103**)	0.29 (0.363)	0.33 (0.059)
	α	-	0.29 (0.518)	0.15 (0.455)	0.13 (0.685)	0.08 (0.639)
	$1/R_{0}$	1/Ω	0.14 (0.764)	**0.61** (**0.001**)	**0.75** (**0.005**)	0.33 (0.061)
	$1/R_{\infty }$	1/Ω	0.02 (0.968)	**0.63** (**0.001**)	**0.73** (**0.007**)	0.41 (**0.017**)
$Q_{2}$ (cm)	$1/R_{ICW}$	1/Ω	0.35 (0.442)	0.45 (**0.020**)	**0.53** (0.077)	0.39 (**0.024**)
	$C_{M}$	*F*	**0.79** (**0.033**)	**0.52** (**0.007**)	**0.62** (**0.032**)	**0.53** (**0.002**)
	τ	*s*	**0.84** (**0.017**)	0.32 (0.106)	0.35 (0.264)	0.46 (**0.007**)
	α	-	0.42 (0.341)	0.20 (0.318)	0.21 (0.521)	0.13 (0.481)
	$1/R_{0}$	1/Ω	0.48 (0.283)	0.08 (0.677)	0.14 (0.657)	0.02 (0.903)
	$1/R_{\infty }$	1/Ω	0.47 (0.295)	0.13 (0.543)	0.24 (0.446)	0.06 (0.721)
$Q_{3}$ (cm)	$1/R_{ICW}$	1/Ω	0.28 (0.553)	0.14 (0.493)	0.26 (0.410)	0.11 (0.553)
	$C_{M}$	*F*	0.10 (0.840)	0.19 (0.342)	**0.53** (0.079)	0.18 (0.320)
	τ	*s*	0.40 (0.375)	0.20 (0.325)	**0.63** (**0.029**)	0.19 (0.281)
	α	-	0.02 (0.965)	0.07 (0.725)	0.01 (0.965)	0.07 (0.713)
	$1/R_{0}$	1/Ω	0.11 (0.807)	0.38 (0.053)	**0.57** (0.053)	0.30 (0.086)
	$1/R_{\infty }$	1/Ω	0.11 (0.822)	0.46 (**0.017**)	**0.62** (**0.032**)	0.40 (**0.020**)
$A_{Q}$ (cm)	$1/R_{ICW}$	1/Ω	**0.52** (0.228)	0.41 (**0.035**)	**0.50** (0.097)	0.41 (**0.017**)
	$C_{M}$	*F*	**0.81** (**0.028**)	0.43 (**0.027**)	**0.57** (0.055)	0.45 (**0.009**)
	τ	*s*	**0.66** (0.104)	0.29 (0.145)	0.38 (0.222)	0.29 (0.102)
	α	-	0.01 (0.990)	0.27 (0.179)	0.27 (0.392)	0.25 (0.172)
	$1/R_{0}$	1/Ω			0.45 (0.143)	
	$1/R_{\infty }$	1/Ω			**0.68** (**0.015**)	
$F_{\mathrm{isoT}}$ (kg)	$1/R_{ICW}$	1/Ω			**0.70** (**0.011**)	
	$C_{M}$	*F*			**0.62** (**0.032**)	
	τ	*s*			0.24 (0.446)	
	α	-			0.16 (0.629)	
	$1/R_{0}$	1/Ω			0.47 (0.124)	
	$1/R_{\infty }$	1/Ω			**0.73** (**0.007**)	
$F_{\mathrm{isoM}}$ (kg)	$1/R_{ICW}$	1/Ω			**0.77** (**0.004**)	
	$C_{M}$	*F*			**0.70** (**0.011**)	
	τ	*s*			0.34 (0.276)	
	α	-			0.29 (0.354)	

**Table 5:** Prediction equation for structural and functional parameters of the quadriceps muscle. ME: Mean error; MSE: Mean square error; r: correlation coefficient; p: statistical significance; N: number of measurements; MD ± CI: Bland–Altman, mean difference ±1.96 times the standard deviation (confidence interval). Results with a correlation greater than 0.7 or a statistical significance less than 0.01 have been bolded.

Prediction Equation	Unit	ME	MSE	r	*p*	N	MD ± CI	Group
$Q_{T}=0.034\cdot C_{M}+1.7$	cm	0.1	0.2	0.68	0.092	7	−0.0±0.7	I
$Q_{T}=0.04\cdot C_{M}+1.4$	cm	0.2	0.2	0.69	<$5\cdot 10^{-4}$	26	0.0±0.9	II
$Q_{T}=0.05\cdot C_{M}+1.1$	cm	0.2	0.2	**0.79**	**0.002**	12	0.0±0.8	III
$Q_{T}=0.039\cdot C_{M}+1.5$	cm	0.2	0.2	0.69	<$5\cdot 10^{-4}$	33	0.0±0.9	Global
$Q_{T}=69/R_{0}+1.3$	cm	0.2	0.3	0.47	**0.006**	33	−0.0±1.0	Global
$Q_{T}=129.7/R_{\infty }-0.9$	cm	0.1	0.2	**0.95**	<$5\cdot 10^{-4}$	12	0.0±0.4	III
$Q_{T}=65.4/R_{\infty }+0.8$	cm	0.2	0.3	0.63	<$5\cdot 10^{-4}$	33	−0.0±0.9	Global
$Q_{T}=148.1/R_{ICW}+1.3$	cm	0.2	0.2	0.65	<$5\cdot 10^{-4}$	33	−0.0±0.9	Global
$Q_{T}=0.00021\cdot \tau +1.5$	cm	0.2	0.3	0.42	0.016	33	−0.0±1.1	Global
$Q_{1}=0.015\cdot C_{M}+0.8$	cm	0.1	0.1	0.47	0.283	7	0.0±0.5	I
$Q_{1}=0.018\cdot C_{M}+0.8$	cm	0.1	0.2	0.55	**0.004**	26	−0.0±0.6	II
$Q_{1}=0.02\cdot C_{M}+0.6$	cm	0.1	0.1	0.67	0.018	12	0.0±0.5	III
$Q_{1}=0.018\cdot C_{M}+0.8$	cm	0.1	0.2	0.53	**0.001**	33	−0.0±0.6	Global
$Q_{2}=0.044\cdot C_{M}+2.7$	cm	0.1	0.2	**0.79**	0.033	7	0.0±0.6	I
$Q_{2}=0.036\cdot C_{M}+2.3$	cm	0.3	0.4	0.52	**0.007**	26	0.0±1.3	II
$Q_{2}=0.04\cdot C_{M}+1.9$	cm	0.2	0.3	0.62	0.032	12	0.0±1.1	III
$Q_{2}=0.038\cdot C_{M}+2.4$	cm	0.3	0.4	0.53	**0.002**	33	0.0±1.3	Global
$A_{Q}=0.052\cdot C_{M}+3.9$	cm^2^	0.2	0.3	**0.81**	0.028	7	0.0±0.7	I
$A_{Q}=0.068\cdot C_{M}+3.6$	cm^2^	0.6	1.1	0.43	0.027	26	−0.0±3.0	II
$A_{Q}=0.108\cdot C_{M}+2.4$	cm^2^	0.7	1.3	0.57	0.054	12	−0.0±3.3	III
$A_{Q}=0.065\cdot C_{M}+3.7$	cm^2^	0.5	1.0	0.45	**0.009**	33	−0.0±2.7	Global
$A_{Q}=106.51/R_{\infty }+2.7$	cm^2^	0.5	1.0	0.40	0.020	33	−0.0±2.7	Global
$A_{Q}=238.37/R_{ICW}+3.4$	cm^2^	0.5	1.0	0.41	0.017	33	−0.0±2.7	Global
$F_{isoT}=862.12/R_{\infty }+6.3$	kg	1.8	3.1	0.68	0.016	12	0.0±9.1	III
$F_{isoT}=1425.43/R_{ICW}+16.1$	kg	2.0	2.9	**0.70**	0.011	12	−0.0±8.9	III
$F_{isoT}=0.36\cdot C_{M}+19$	kg	2.1	3.1	0.62	0.032	12	0.0±9.7	III
$F_{isoM}=2160.16/R_{\infty }+1.3$	kg	4.2	9.5	**0.73**	**0.007**	12	−0.0±19.7	III
$F_{isoM}=3624.76/R_{ICW}+25.6$	kg	3.9	7.0	**0.77**	**0.004**	12	−0.0±18.5	III
$F_{isoM}=0.96\cdot C_{M}+32$	kg	4.5	8.6	**0.70**	0.011	12	−0.0±20.5	III

## Data Availability

The data presented in this study are available on request from the corresponding author.
